# Sleep, light exposure at night, and psychological wellbeing during pregnancy

**DOI:** 10.1186/s12889-023-16655-y

**Published:** 2023-09-16

**Authors:** Choon Ming Ng, Satvinder Kaur, Ee Yin Kok, Wan Ling Chew, Masaki Takahashi, Shigenobu Shibata

**Affiliations:** 1 School of Pharmacy, Monash University Malaysia, South Lagoon Road, 47500 Bandar Sunway, Selangor Darul Ehsan Malaysia; 2 Faculty of Applied Sciences, UCSI University, 1, Jalan Puncak Menara Gading, Taman Connaught, 56000 Kuala Lumpur, Malaysia; 3 Institute for Liberal Arts, Tokyo Institute of Technology, 2-12-1 Ookayama, Meguro-ku, Tokyo, 152-8550 Japan; 4 Graduate School of Biomedical and Health Sciences, Hiroshima University, 1-2-3 Kasumi, Minami-ku, Hiroshima City, Hiroshima, 734-8551 Japan

**Keywords:** Sleep, Light, Stress, Anxiety, Depression, Pregnancy

## Abstract

**Background:**

Psychological wellbeing during pregnancy is imperative for optimal maternal outcomes. The present study aimed to determine the association between sleep quality, light exposure at night, and psychological wellbeing in the 2^nd^ and 3^rd^ trimesters of pregnancy.

**Methods:**

This prospective study was conducted in 9 randomly selected government maternity clinics in Kuala Lumpur, Malaysia. Healthy women aged 20–48 years old with single pregnancy were recruited using convenience sampling (*n* = 169). Sleep quality, light exposure at night, and psychological wellbeing were self-reported using the Pittsburgh Sleep Quality Index (PSQI), Harvard Light Exposure Assessment (H-LEA), and Depression, Anxiety, and Stress Scale (DASS-21) in the 2^nd^ trimester and followed-up at the 3^rd^ trimester.

**Results:**

During the 2^nd^ and 3^rd^ trimesters of pregnancy, mild to severe symptoms of stress (10.7 and 11.3%), anxiety (42 and 44.3%), and depression (9.6 and 16.6%) were observed among the participants. Adjusted multiple linear regression revealed that poor sleep quality and higher light exposure at night were attributed to greater stress and depression symptoms in the 3^rd^ trimester. Higher lux level exposed from 10 pm to < 1 am was associated with increased stress (β = 0.212, *p* = 0.037) and depression (β = 0.228, *p* = 0.024). Only poor sleep quality was observed to adversely affect anxiety (β = 0.243, *p* = 0.002) and depression levels (β = 0.259, *p* = 0.001) in the 2^nd^ trimester.

**Conclusions:**

Present study provided preliminary findings on the association between sleep quality, light at night, and psychological wellbeing of pregnant women. As a recommendation, future research could investigate whether public health interventions aimed at decreasing artificial light at night can benefit sleep quality and the psychological health of pregnant women.

## Background

Psychological wellbeing is the concept of optimal mental functioning that improves the overall health of individuals. Compromised psychological wellbeing continues to be a prevalent issue faced by pregnant women in countries worldwide, with issues such as heightened stress, anxiety, and depression symptoms. Globally, the prevalence of stress, anxiety, and depression among pregnant women ranged between 23–56.8%, 37.5–63%, and 19.9–45.2`% respectively [1–3]. Pregnant women are especially vulnerable to psychological issues as pregnancy itself is a process where major biological, hormonal, and life changes occur [4]. The focus on maternal psychological wellbeing is imperative since it relates closely to birth outcomes and infant development through fetal programming. For instance, poor psychological wellbeing in pregnant mothers was associated with preterm birth, low birth weight babies, and impaired fetal brain development [5].

Throughout the years, research has documented numerous factors that play critical roles in affecting psychological wellbeing during pregnancy. Particularly, sleep, a biological process for physiological wellness, was extensively studied. Past studies elucidated that poor sleep quality, short sleep duration, and sleep disturbances were linked with negative psychological outcomes among pregnant women [6, 7]. Moving forward, research continued to reveal the complexity of the relationship between sleep and psychological wellbeing, to which additional variables could have an influence. Recently, light exposure has been linked to psychological wellbeing in various populations, along with sleep as a possible influence [8]. The relationship between light and sleep as factors affecting psychological wellbeing could be attributed to their close connection to circadian rhythm regulation.

Undoubtedly, artificial light usage has increased tremendously with modernization and technological advancement, especially at night. As the main exogenous zeitgeber to the body’s internal clock, light influences the sleep–wake cycle and circadian rhythm. When light reaches the eye and the retina, it is detected by the retinal non-image forming photoreceptors and in turn, the light perceived is transmitted to the master clock suprachiasmatic nuclei (SCN) neurons [9]. Subsequently, the electric signals from the SCN activate several signaling pathways that affect vital body processes in accordance with the natural 24-h light–dark cycle, including melatonin production [10].

Although artificial light at night has brought convenience to our lives, improper/excessive exposure to artificial light out of sync from the natural light–dark cycle may bring about negative consequences including circadian misalignment that suppressed melatonin and other physiological processes, increased risk of sleep disorders, and compromised mental health [10]. For example, nationally-representative data illustrated that increased outdoor artificial lighting measured via satellite impacted adolescents’ sleep health negatively and was associated with higher prevalence of anxiety and other mood disorders [11]. Besides, research evidence demonstrated the harmful effects of light exposure at abnormal timings on mental wellbeing among shift workers [12]. Furthermore, research has shown that stress levels were elevated among children exposed to artificial light at night, whereby sleep acts as a mediator in the Eq. [13]. Among the elderly population, exposure to light at night was associated with depressive symptoms when sleep disturbances were controlled in the study [14].

While the relationship between light exposure and psychological wellbeing is not fully understood yet, a plausible mechanism was proposed including the effect of light on circadian system, which in turn, affects important biological rhythms in the body that may affect mood-related behaviours during pregnancy [11]. The close connection between light and psychological wellbeing can also be observed in the use of light therapy as a treatment for mood disorders, including during pregnancy [15–17]. Yet, the relationship between light, sleep, and psychological wellbeing during pregnancy remained understudied and further exploration is much needed. Particularly in the context of pregnant women, most studies assessing sleep quality and psychological wellbeing did not take light exposure into consideration, which could possibly affect study outcomes as shown in other populations aside from pregnant women.

Interestingly, a recent study has done a comparison of sleep quality and perceived stress, accounting for light exposure before bedtime among pregnant and non-pregnant women [18]. However, only perceived stress was measured in the said study as an indicator of poor psychological wellbeing. The spectrum of psychological wellbeing can be further expanded by considering feelings of despair and excessive worry to present more comprehensive data. Moreover, the study results also showed no associations between sleep quality and psychological wellbeing, contradicting previous literature. One of the reasons could be related to the said study setting in Norway, an economically stable country where participants tend to display low stress levels. As highlighted earlier [19], it is critical to explore the risk factors for poor psychological wellbeing, including underexplored populations in the Asia region like Malaysia.

Although past studies had investigated sleep quality and its association with psychological wellbeing among pregnant women throughout 2^nd^ and 3^rd^ trimesters [20], the role of light exposure is often understudied. As such, the present study aimed to determine the association between sleep quality, light exposure at night, and psychological wellbeing among women throughout 2^nd^ and 3^rd^ trimesters of pregnancy. Study findings would allow for the identification of trends and critical timing during pregnancy for interventions to be implemented.

## Methods

### Participants

This was a prospective cohort study conducted in the 2nd trimester and followed up during the 3^rd^ trimester. Data collection was conducted from July 2019 to April 2021. Pregnant women were recruited from 9 government maternity clinics in Kuala Lumpur, Malaysia, selected using simple random sampling. The participants were recruited using a convenience sampling method during their antenatal check-ups at the selected maternal clinics. Those who were Malaysians aged between 20–48 years old and literate in English or Bahasa Malaysia were recruited for the study. A minimum sample size required was 69 participants, calculated based on the probability of poor sleep quality among pregnant women with (*p* = 0.18) and without antenatal depression (*p* = 0.39), with confidence level at 95% and 80% power [20]. After excluding pregnant women with multiple pregnancies, shift workers, and those suffering from serious health conditions such as gestational diabetes, hypertension, pre-eclampsia, or anaemia, 319 eligible pregnant women were approached for the study, of which 169 agreed to participate and completed the assessment in the 2^nd^ trimester (53% success in the study recruitment). Reasons for declining participation include lack of time and lack of support from family attributing to the commitment required for the research, particularly during the critical period of pregnancy. A total of 115 follow-up data was collected during the 3rd trimester, giving rise to a 31.9% loss mainly due to miscarriage, loss of contact due to unknown reasons, and movement control order related to Covid-19 pandemic where residents were encouraged to stay at home for non-essential activities. While online options were provided during the pandemic, some declined to participate due to the other negative implications related to the pandemic, as well as a lack of digital literacy.

All procedures performed were in accordance with the ethical standards of the Declaration of Helsinki. All experimental protocols were approved by the Medical Research and Ethics Committee (KKM/NIHSEC/P19-125) and National Medical Research Registrar (NMRR-18–3412-45225), to which ethical standards set by the committee were met. Approval letter from the Health Department of Kuala Lumpur and Putrajaya was obtained to conduct research in government maternity clinics. Besides, a written informed consent form was collected from the participants prior to data collection.

## Measures

### Sociodemographic and health records

In the maternity clinic, sociodemographic details (age, household income) were collected via self-administered questionnaires. Medical history such as previous birth complications and physical impairments were cross-checked with antenatal health records.

### Sleep quality

The sleep quality of pregnant women was measured using the Pittsburgh Sleep Quality Index (PSQI), which evaluated the sleep quality for the past month [21]. PSQI is a validated questionnaire that measures 7 derived components of sleep quality: subjective sleep quality, sleep latency, habitual sleep efficiency (%), sleep duration (hours), sleep disturbances, use of sleep medication, and daytime dysfunction. Each subdomain was scored from 0 to 3, where a higher value corresponded to more severe sleep difficulties. The summed scores from 7 subdomains were produced to calculate the total global PSQI score, ranging from 0 to 21. A total global score of more than 5 indicated poor sleep quality, while a total global score of less than 5 reflected good sleep quality.

### Light exposure

Harvard Light Exposure Assessment (H-LEA) assessed light exposure at each hour for 3 consecutive days with 2 weekdays and 1 weekend day [22]. The questionnaire groups light into several sources: (1) artificial light including i) halogen lamp, ii) fluorescent lamp, iii) incandescent light, iv) light-emitting diodes (LED) lamp, v) other artificial light such as television, tablet, smartphone, computer, and candlelight, (2) natural light such as i) indoor natural light, ii) outdoor natural light, and lastly, (3) darkness. The participants were required to identify the type of light sources for each hour in the questionnaire. The light sources at each timing were converted into their respective lux levels, corresponding to the light intensity of surfaces in indoor and outdoor sources. Light at night was defined as light exposure from 7 pm to 7 am (based on Malaysia’s sunrise and sunset time) and recorded according to time blocks [23]. Time was categorized into 3-h intervals: 7 pm to  < 10 pm, 10 pm to  < 1 am, 1 am to  < 4 am, and 4 am to  < 7 am. The average lux levels for 3 days were determined and grouped into their respective time intervals for 2^nd^ and 3^rd^ trimesters of pregnancy.

### Psychological wellbeing

Depression, Anxiety, and Stress Scale – 21 (DASS-21) was used to assess the levels of depression, anxiety, and stress of pregnant women over the past week [24]. The questionnaire consists of 7 items for each component: depression, anxiety, and stress. Participants were required to rate their condition according to a 4-point Likert scale of 0 (did not apply to me at all) to 3 (applied to me very much or most of the time) for the past week. The scores for each subscale were then summed up and multiplied by 2 to get the final score. The participant’s psychosocial wellbeing was categorized based on the severity of each component. The cut-off values for depression were normal (0–9), mild (10–13), moderate (14–20), and severe (≥ 21). For anxiety symptoms, the corresponding cut-off values were normal (0–7), mild (8–9), moderate (10–14), and severe (≥ 15). The suggested cut-off scores for stress symptoms were normal (0–14), mild (15–18), moderate (19–25), and severe (≥ 26).

### Statistical analyses

Statistical analysis was performed using SPSS version 23 software (IBM, USA). The normality of data distribution was determined using the Shapiro Wilk test. Continuous variables were presented in mean ± standard deviation, while categorical variables were presented as frequency (percentage). We performed a paired samples t-test to compare sleep quality, psychosocial wellbeing, and light exposure (lux levels) among pregnant women in their 2^nd^ and 3^rd^ trimesters. The association between lux levels and sleep quality with psychological wellbeing during pregnancy was then evaluated using multiple linear regression adjusted for maternal age and household income. The observed association was expressed as beta coefficient (β) corresponding to 95% confidence intervals (CI). The effect size for paired samples t-test (Cohen’s d) and multiple linear regression analysis (R^2^, adjusted R^2^, Cohen’s f^2^) were assessed accordingly.

## Results

The characteristics of participants were presented in Table 1.

**Table 1:** Characteristics of participants (*n* = 169)

Characteristics	n (%)
Maternal age^a^	28.2 (3.8)
Monthly household income^b^	
Low income (< RM2300)	27 (16.0)
Middle income (RM 2300 – 5599)	95 (56.2)
High income (> RM5600)	47 (27.8)

*Abbreviations*: *SD* standard deviation

^a^Data reported as mean (SD)

^b^Based on 10th Malaysia Economic Plan

As pregnant women progressed from the 2^nd^ to 3^rd^ trimester, their sleep quality deteriorated (Table 2). This can be observed based on the global PSQI score (5.7 to 6.2; *p* = 0.046), sleep disturbance (9.9 to 11.1; *p* = 0.002), and sleep duration score (0.9 to 1.1; *p* = 0.026) with small yet non-trivial effects.

**Table 2:** Sleep Quality at 2nd and 3rd Trimesters (*n* = 115)

**Variables**	**Mean (SD)**		**Cohen’s d**	***p*-value**
	**2**^**nd**^** trimester**	**3**^**rd**^** trimester**		
Global PSQI score	5.7 (2.6)	6.2 (2.9)	0.18	0.046*
Subjective sleep quality	1.0 (0.62)	1.1 (0.62)	0.16	0.116
Sleep latency	1.8 (1.7)	1.9 (1.6)	0.06	0.407
Sleep duration	0.9 (0.9)	1.1 (0.9)	0.20	0.026*
Sleep efficiency (%)	87.9 (12.9)	86.6 (12.4)	0.10	0.375
Sleep medication	0.04 (0.30)	0.01 (0.09)	0.15	0.250
Daytime dysfunction	1.0 (1.0)	0.9 (1.1)	0.10	0.379
Sleep disturbance	9.9 (4.4)	11.1 (4.4)	0.27	0.002*

*Abbreviations*: *PSQI* Pittsburgh Sleep Quality Index. *SD* standard deviation

Data reported as mean (SD) and p-value reported according to paired-sample t-test analysis

**Fig. 1:**
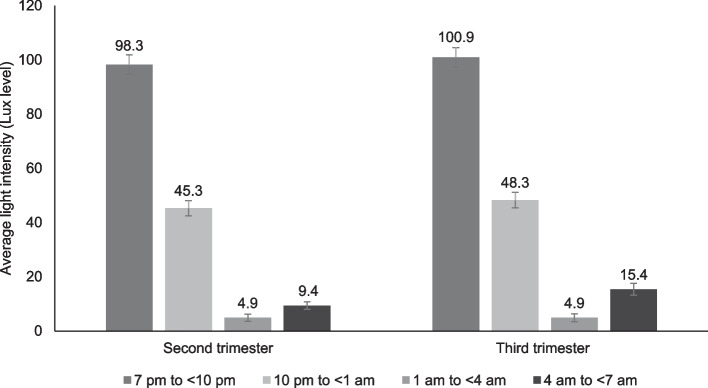
Lux Level at 2nd and 3rd Trimesters (*n* = 115). Paired samples t-test showed a significant difference in lux level (4 am to < 7 am) from the 2nd to 3rd trimester (Cohen’s d = 0.32, *p* = 0.005)

In terms of light exposure at night, the highest lux exposed was at 7 pm to < 10 pm, followed by 10 pm to < 1 am, and the lowest exposure at sleep time 1 am to < 4 am (Fig. 1). When nearing dawn and sunrise, the lux level slightly increased at 4 am to < 7 am. Similar patterns were observed in the 2^nd^ and 3^rd^ trimesters. However, it should be noted that the lux level generally increased from the 2^nd^ to the 3^rd^ trimester at all time blocks, despite insignificant increases from 7 pm to < 4 am. Moreover, the lux level exposed at the time block 4 am to < 7 am was significantly higher in the 3^rd^ trimester (15.4 vs 9.4 in the 2^nd^ trimester, *p* = 0.05), with a small non-trivial effect size.

**Fig. 2:**
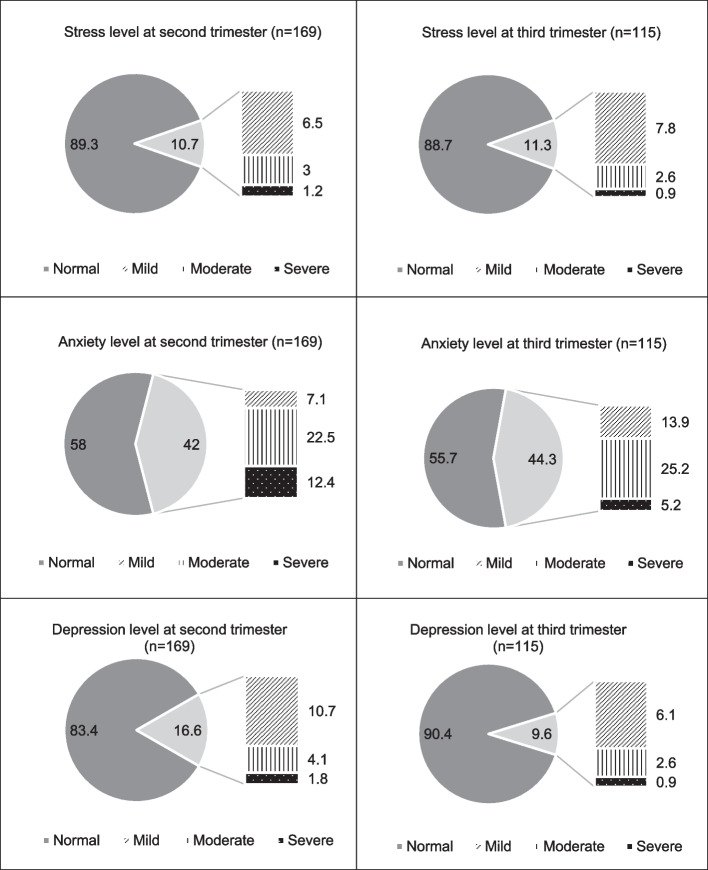
Psychological Wellbeing of Pregnant Women at 2nd and 3rd Trimesters. Paired samples t-test showed no significant difference in psychological wellbeing from 2nd to the 3rd trimester

Furthermore, no significant difference was observed in the psychological wellbeing of pregnant women in the 2^nd^ and 3^rd^ trimesters (Fig. 2). Nevertheless, stress, anxiety, and depression were prevalent conditions in some pregnant women. Approximately one in ten pregnant women exhibited mild to severe symptoms of stress (ranging from 10.7–11.3% of the pregnant women) and depression (ranging from 9.6–16.6% of the pregnant women). More importantly, it should be highlighted that almost half of the pregnant women displayed mild to severe symptoms of anxiety in both trimesters (ranging from 42% to 44.3%).

Adjusted multiple linear regression revealed that sleep quality measured by global PSQI score and lux level were consistently attributed to greater stress and depression symptoms in the 3^rd^ trimester, with medium effect sizes observed (Table 3). Specifically, higher lux level exposed at 10 pm to < 1 am was associated with increased stress (β = 0.212, *p* = 0.037) and depression (β = 0.228, *p* = 0.024). On the other hand, there was a lack of association between lux level and the psychological wellbeing of pregnant women in the 2^nd^ trimester (Table 4). Only poor sleep quality was observed to adversely affect anxiety (β = 0.243, *p* = 0.002) and depression levels (β = 0.259, *p* = 0.001) in the 2^nd^ trimester with small yet non-trivial effects.

**Table 3:** Associations between lux level and sleep quality with psychological wellbeing during the 3rd trimester of pregnancy (*n*=115)^a^

Variables	Stress^b^		Anxiety^c^		Depression^d^	
	β (95% CI)	*p*-value	β (95% CI)	*p*-value	β (95% CI)	*p*-value
Global PSQI score	0.290 (1.410, 6.059)	0.002*	0.247 (0.738, 4.859)	0.008*	0.305 (1.133, 4.530)	0.001*
Lux level						
7 pm-10 pm	-0.112 (-0.045, 0.013)	0.268	-0.111 (-0.042, 0.012)	0.265	-0.080 (-0.031, 0.013)	0.425
10 pm-1 am	0.212 (0.002, 0.076)	0.037*	0.161(-0.006, 0.062)	0.106	0.228 (0.004, 0.060)	0.024*
1 am-4 am	0.025 (-0.064, 0.082)	0.807	0.195 (0.000, 0.135)	0.050	0.065 (-0.037, 0.074)	0.512
4 am-7 am	-0.045 (-0.059, 0.037)	0.655	0.028 (-0.038, 0.051)	0.776	-0.092 (-0.054, 0.019)	0.352

*Abbreviations*: *PSQI* Pittsburgh Sleep Quality Index, *95% CI* 95% Confidence Intervals

*p-value significant at <0.05

^a^Adjusted for maternal age and household income

^b^Model significant at *p* = 0.045. *R* = 0.368, *R*^2^ = 0.135, Adjusted *R*^2^ = 0.070, Cohen’s f^2^ = 0.16

^c^Model significant at *p* = 0.014. *R* = 0.402, *R*^2^ = 0.162, Adjusted *R*^2^ = 0.099, Cohen’s f^2^ = 0.19

^d^Model significant at *p* = 0.022. *R* = 0.389, *R*^2^ = 0.151, Adjusted *R*^2^ = 0.087, Cohen’s f^2^ = 0.18

**Table 4:** Associations Between lux level and sleep quality with psychological wellbeing during the 2nd trimester of pregnancy (*n*=169)^a^

Variables	Stress^b^		Anxiety^c^		Depression^d^	
	β (95% CI)	*p*-value	β (95% CI)	*p*-value	β (95% CI)	*p*-value
Global PSQI score	-	-	0.243 (1.092, 4.880)	0.002*	0.259 (1.092, 4.880)	0.001*
Lux level						
7 pm- 10 pm	-	-	0.044 (-0.019, 0.032)	0.620	-0.034 (-0.028, 0.019)	0.699
10 pm-1 am	-	-	0.096 (-0.013, 0.050)	0.250	0.113 (-0.009, 0.049)	0.180
1 am-4 am	-	-	0.049 (-0.037, 0.061)	0.632	-0.140 (-0.078, 0.015)	0.177
4 am-7 am	-	-	0.071 (-0.009, 0.018)	0.486	0.049 (-0.010, 0.016)	0.632

*Abbreviations*: *PSQI* Pittsburgh Sleep Quality Index, *95% CI* 95% Confidence Intervals

*p-value significant at <0.05

^a^Adjusted for maternal age and household income

^b^Model insignificant at *p* = 0.202. *R* = 0.246, *R*^2^ = 0.061, Adjusted *R*^2^ = 0.014, Cohen’s f^2^ = 0.06

^c^Model significant at *p* = 0.009. *R* = 0.344, *R*^2^ = 0.118, Adjusted *R*^2^ = 0.074, Cohen’s f^2^ = 0.13

^d^Model significant at *p* = 0.037. *R* = 0.309, *R*^2^ = 0.096, Adjusted *R*^2^ = 0.050, Cohen’s f^2^ = 0.11

## Discussion

In the current study, we assessed the psychological wellbeing, sleep quality, and light exposure level at night in 2^nd^ and 3^rd^ trimesters. As reported earlier [25], stress, anxiety, and depression were relevant conditions present in pregnant women. Given that stress, anxiety, and depression are risk factors for adverse maternal and family outcomes, it is crucial to determine the possible issues associated with pregnant women exhibiting such symptoms. In the present study, light exposure and sleep were both explored as factors affecting psychological wellbeing. The rationale is that light acts as an external cue for the circadian pacemaker and hence, plays a role in key biological processes including the production and secretion of melatonin via the retina-hypothalamic pathways [26]. Indeed, light exposure serves as a zeitgeber to consolidate sleep-wakefulness cycles, often affecting sleep onset and quality [27].

Current study findings showed the associations of light exposure and sleep with psychological wellbeing during pregnancy in the 3^rd^ trimester of pregnancy. Undoubtedly, the relationship between sleep, light, and psychological wellbeing is complex, and it is not possible to establish the causal effect based on the study design. However, several hypotheses could be raised based on the findings. Firstly, light exposure at night could adversely affect the circadian rhythm [10], which regulates numerous physiological functions including mood regulation through transcriptional and translational feedback loops [9]. Environmental disruptions such as light can exacerbate the desynchronization of the rhythms, influencing processes involved in mood regulation. In this regard, several mechanisms have been put forth regarding the influence of synchronized circadian rhythm and its disruptions on mood regulation. These include involvement in monoamine signaling in the brain region that is responsible for mood and anxiety-related behaviors through clock gene expressions, altered immune function with increased pro-inflammatory cytokines associated with circadian disruption, as well as the hypothalamus–pituitary–adrenal axis regulation that stabilizes mood [28].

Several studies conducted thus far could help shed light on the relation between light exposure, circadian misalignment, and subsequently, poor psychological wellbeing. First, it has been shown that approximately 6.5 h exposure to 100 lx level at night can produce one-half of the response for a stimulus almost 100 times brighter (9000 lx), suggesting that the circadian rhythm is highly sensitive to light intensity and duration exposed, thereby generating significant phase-delaying effect and melatonin suppression [29]. A preliminary study among women with a history of major depressive disorder found that delayed circadian phase shift measured by dim light salivary melatonin at 6 weeks postpartum relative to the third trimester of pregnancy was associated with depressive mood [30]. Further, the disturbances to biological rhythms measured objectively and subjectively, among those with and without history of mood disorders were linked to poor psychological wellbeing during the perinatal period [31–33].

In terms of wavelengths of light, a recent randomized-controlled trial among pregnant women elucidated that the use of blue-blocking glass 3 h before bedtime had favorable effect on the circadian rhythm with advanced melatonin onset and increased melatonin levels at night [34]. Promisingly, the application of bright light therapy for improving psychological wellbeing particularly depression during pregnancy has been reported. For instance, multiple studies demonstrated that bright light therapy in the morning improved depression among pregnant women as compared to the control group [7000 lx vs 70 lx [17], 7000 lx vs 500 lx [16]]. Proper light exposure, could regulate mood by producing inputs to the perihabenular nucleus of the dorsal thalamus which projects to mood-regulating centers [35].

On the other hand, light exposure at night could act as a stressor to pregnant women which disrupted sleep and mood, contributing to the feelings of stress. For instance, when healthy participants were exposed to light (100 lx), subjective sleep quality and rapid eye movement (REM) sleep decreased, while light sleep and sleep awakening increased with lower nocturnal melatonin level and poorer psychological wellbeing [36, 37]. In addition, a trend of increased cortisol (stress hormone) was invoked in those exposed to light at night, although this was insignificant between groups, possibly due to the low sample size [36]. In another experimental study among hospitalized patients, a dynamic 24-h light–dark cycle with minimal light exposure at night and 2 h of bright light (1750 lx) during the day had better objective sleep measures as compared to standard fluorescent lighting throughout the day and night [38]. Despite that, no significant difference was seen in anxiety and depression symptoms between groups, possibly since both groups had ‘normal’ scores, leaving little room for improvement. Meanwhile in healthy adults, moderately bright light (1000 lx) in the morning boosted subjective mood, motivation, and happiness with reduced feelings of anxiety [39]. A recent epidemiology study elucidated that satellite images of increased outdoor artificial light at night were associated with poor sleep patterns, mood, and depressive symptoms in young adults [11]. Among elderly adults, exposure to lux levels of more than 5 lx at bedtime to rising time exhibited a significantly greater depression risk after 24 months [40].

Recently, the American Medical Association issued a policy statement based on mounting research evidence on the effect of artificial light on human physiology with direct connections to health, including mental wellbeing, mood, and depression [26]. True to our findings, it seems that the state of good mental health is promoted through a mechanism involving internal clocks alignment to the natural dark–light cycle, without excessive exposure to light at night.

In terms of sleep quality, the global PSQI score of participants in the present study was comparable with a recent meta-analysis comprising 24 studies and a total of 11,022 pregnant women. The meta-analysis reported an average score of 6.09 [41], corresponding to poor sleep quality (PSQI ≥ 5 points). Likewise, the sleep quality of pregnant women deteriorated from 2^nd^ to 3^rd^ trimester in the current study, often reported in longitudinal studies [41]. Such findings suggest that poor sleep quality continues to be a common issue faced by pregnant women, especially as it progresses to the final trimester of pregnancy.

Our findings add to the body of literature on the association between negative sleep quality and poor mental health in pregnant women [20]. The relationship between sleep and psychological wellbeing has been proposed as bidirectional, with poor sleep quality resulting from suboptimal emotional health and concurrently regarded as physiological stressors per se, leading to feelings of overload [42]. Any possible scenarios that add to the feelings of stress can result in the activation of allostasis (maintenance of homeostasis through adaption processes) and contribute to allostatic load (effects of repeated burden of stress on body) [43], affecting sleep outcomes. For instance, it was reported that allostatic load in pregnant women was positively correlated with poor sleep quality, as measured by PSQI [44]. Subsequently, disturbed sleep quality can result in a continuous cycle of persistent inflammatory responses, generating allostatic load and contributing to poor mental health such as symptoms of depression [42, 45].

The present study has several limitations to note. Owing to our study design, it is possible that those who exhibit symptoms of stress and depression tend to sleep late with increased exposure to light at night. Further, we did not account for light exposure throughout the day, precluding a comprehensive characterization of 24-h light exposure, which could affect sleep outcomes and psychological wellbeing [46]. Additionally, sleep quality and light exposure were assessed through self-reported means and thus, could be subjected to recall and social desirability bias. Nonetheless, the confidentiality of the data was assured to the pregnant women. While H-LEA allows the identification of light sources and lux levels, the H-LEA assessed light exposure subjectively, and hence, objective measures will be useful for further exploration in future research. Additionally, the DASS-21 scale is designed to assess signs of poor psychological wellbeing, yet it is inadequate for identifying and diagnosing the various types of depressions such as major depressive disorder, persistent depressive disorder, bipolar disorder, seasonal affective disorder, chronic or recurrent depression. Of note, our analyses elucidated that lux level exposed and sleep had non-trivial, weak to moderate effect sizes on psychological wellbeing. In regards to this, it is important to note that the majority of participants assessed (~ 83–90%) did not show symptoms of stress and depression in the 2^nd^ and 3^rd^ trimesters of pregnancy, while more than half did not exhibit anxiety symptoms, thereby limiting potential associations. Furthermore, we found no significant association between light exposure in the 2nd trimester with psychological wellbeing in 3rd trimester (analyses not shown). It seemed that the influence of light on psychological wellbeing is acute in the present preliminary findings and perhaps, longer exposure, follow-up periods, and objective assessments would provide better insights. We also did not assess possible comorbidity with other conditions such as substance use disorders and the link with light exposure as this was beyond the scope of current study. In addition, the study was conducted in the capital of Malaysia, Kuala Lumpur. While we did not recruit pregnant women from all states in Malaysia, Kuala Lumpur remained one of the most populous and fastest-growing cities in the country, reflecting the multicultural nation with citizens originating across all states in Malaysia [47]. Nevertheless, the study findings must be interpreted with caution given that it was conducted in one state of Malaysia, and thus, may not be generalizable in other settings. Lastly, we faced common barriers to study retention especially related to the degree of inconvenience imposed on the participants as reported previously [48, 49], and we acknowledged that the loss to follow-up may affect present findings. Despite that, we conducted further analysis of participant’s characteristics and found no significant difference between those who dropped out and those who were retained in the study. We also ensured that the final sample size was sufficiently powered.

In conclusion, the study provided preliminary findings that light exposure at night, sleep quality, and psychological wellbeing were closely linked. Study findings highlighted the need to improve sleep quality and to boost lighting strategies for the promotion of emotional care during the critical period of pregnancy. In turn, this could have long-term implications on maternal and child outcomes. As a recommendation, future research could investigate whether public health interventions aimed at decreasing artificial light at night can benefit sleep quality and the psychological health of pregnant women.

## Data Availability

The datasets used and/or analysed during the current study are available from the corresponding author upon reasonable request.

## Abbreviations

DASS-21: Depression Anxiety and Stress Scale
CI: Confidence interval
H-LEA: Harvard Light Exposure Assessment
PSQI: Pittsburgh Sleep Quality Index
SD: Standard deviation
